# Tertiary-Amine Functionalized Polyplexes Enhanced Cellular Uptake and Prolonged Gene Expression

**DOI:** 10.1371/journal.pone.0097627

**Published:** 2014-05-14

**Authors:** Chia-Wen Lo, Yung Chang, Jyun-Lin Lee, Wei-Bor Tsai, Wen-Shiang Chen

**Affiliations:** 1 Department of Physical Medicine and Rehabilitation, National Taiwan University Hospital and National Taiwan University College of Medicine, Taipei, Taiwan, ROC; 2 R&D Center for Membrane Technology and Department of Chemical Engineering, Chung Yuan Christian University, Jhong-Li, Taoyuan, Taiwan, ROC; 3 Department of Chemical Engineering, National Taiwan University College of Medicine, Taipei, Taiwan, ROC; Universidad de Castilla-La Mancha, Spain

## Abstract

Ultrasound (US) has been found to facilitate the transport of DNA across cell membranes. However, the transfection efficiency is generally low, and the expression duration of the transfected gene is brief. In this study, a tertiary polycation, Poly(2-(dimethylamino) ethyl methacrylate) (PDMAEMA), was used as a carrier for US-mediated gene transfection. Its in-vitro and in-vivo effects on the transfection efficiency and the expression duration were evaluated. A mixture of pCI-neo-luc and PDMAEMA was transfected to cultured cells or mouse muscle by exposure to 1-MHz pulse US. A strong expression of luciferase was found 10 days after the transfection in vitro regardless of US exposure. However, effective transfection only occurred in the US groups in vivo. The transfection ability depended on the weight ratio of PDMAEMA to DNA, and was different for the in-vitro and in-vivo conditions. Lower weight ratios, e.g., 0.25, exhibited better in-vivo expression for at least 45 days.

## Introduction

Gene therapy has been shown to have great potential to overcome the drawbacks associated with conventional drug and protein therapeutic regimens for a range of diseases such as inherited immune deficiencies, cardiovascular disease, and cancer. In the past, viral vectors have been predominantly used for gene therapy due to their ease of use and high efficiency. However, several issues related to safety and immunogenicity have restricted the clinical application of viral vectors [1], [2]. Alternatively, non-viral vectors for gene internalization, such as electroporation, cationic polymer, liposomes, gene gun, hydrodynamics, and ultrasound (US), have been reported to be safer and easier to use than viral vectors [3]. Among the non-viral vectors, US-mediated gene transfer has recently received considerable attention [4]–[9]. Many of the desired characteristics of gene therapy, including low immunogenicity, minimal invasiveness, site specificity, and safety for repeated treatments could be realized by applying the US transfer technique with microbubbles.[8], [10]–[13].

However, along with other non-viral modalities, the US method suffers from low transfection efficiency and a short expression duration for transfected plasmids. It has been reported that, short of optimal exposure conditions, the transfection efficiency could be as low as a few percent of cultured cells accompanied by considerable cell toxicity [14]. Moreover, plasmids are only transiently expressed after conventional US-facilitated transfection, generally peaking at 4–7 days and then decaying exponentially. With rare exceptions, they are essentially gone in three weeks [6], [7].

Polymeric nonviral vectors have been extensively studied for *in vitro* and *in vivo* therapeutic gene delivery [15]. They are gradually considered as more potent than viral vectors and have the additional advantage of lower toxicity and immunogenicity. Previous research has shown that cationic polymers such as polyethylenimine (PEI) could enhance transfection efficiency both *in vitro* and *in vivo* [16], [17]. PEI has a high density of amine groups with positive charges and can bind the negatively-charged DNA molecules to form nanoparticle complexes (polyplexes). It has also been shown to interact with negatively-charged cell membranes and internalize itself into the cell through endocytosis. After crossing the membranes, PEI can act as a buffer to induce osmotic swelling and force the release of PEI/DNA complexes from endosomes, which is essential for their later expression[18]. PEI has also been shown to protect combined DNA from enzyme digestion[19]. However, PEI is toxic under certain conditions, and its transfection efficiency depends on cell types and surface receptors [15], [20]–[23]. Combining US with PEI/DNA nanoparticles has been shown to further enhance transfection efficiency [20], [24]. Our recent study has further shown that PEI could prolong the expression of transfected DNA for at least 30 days, and US exposure is crucial for localized gene transfection on mice muscle using PEI. In certain ways US helps bypass cell type or surface receptor limitations of PEI for *in vivo* conditions [25].

Another polymer which shows promise for facilitating DNA internalization is poly(2-(dimethylamino) ethyl methacrylate) (PDMAEMA). Considerable effort has been expended to understanding the cytotoxicity and transfection efficiency of this type of polycation, both *in vitro* and *in vivo* [26]–[29]. Unlike PEI, PDMAEMA could be synthesized from its monomer, 2-(dimethylamino) ethyl methacrylate or DMAEMA, in a well-controlled living polymerization to produce linear polymers with similarly repetitive units or complex branched structures with higher homogeneity. Furthermore, the pure tertiary amine structure, which is different from the linear form PEI (secondary amine) and the mixed primary, secondary and tertiary amine structure of branched PEI, may possess controllable structures and interesting properties for gene delivery.

To search for a PEI substitute, we synthesized a low molecular weight (6.1 kDa) linear PDMAEMA and systemically evaluated its physical properties, cytotoxicity, and binding ability. The polymers were then evaluated in terms of their ability to transfect BNL cells and mouse thigh muscle with and without the help of US. The differences between PDMAEMA and PEI are also provided.

## Materials and Methods

### Ethics Statement

This study was carried out in strict accordance with the recommendations in the Guide for the Care and Use of National Taiwan University College of Medicine and College of Public Health Institutional Animal Care and Use Committee (IACUC). The protocol was approved by the Institutional Animal Care and Use Committee on National Taiwan University College of Medicine and College of Public Health (Permit Number: 20130061). All surgery was performed under isoflurane anesthesia, and all efforts were made to minimize suffering.

### Preparation of PDMAEMA polycation

N,N-Dimethylaminoethyl methacrylate (DMAEMA) monomers and ammonium persulfate (APS) were purchased from Sigma-Aldrich (St. Louis, MO, USA). A total solids content of 15 wt % for the defined molar ratio of DMAEMA monomer and APS initiator (45/1) was dissolved in 15 mL of deionized water, and nitrogen was bubbled through to remove residual oxygen. The reaction was stirred under positive nitrogen pressure for 6 h at 70°C. After polymerization, the resulting reaction solution was cooled to 4°C for 3 h and then added slowly to n-hexane at 4°C and then repeatedly re-dissolved into deionized water at 25°C to precipitate the polymer out of the reaction solution and to remove residual reagents. The polymer was dried in a freeze-dryer at −45°C to yield a white powder with a molecular weight of 6142 Da and 39 repeat units. The molecular weight of the prepared PDMAEMA polycation was determined by aqueous gel-permeation chromatography (GPC), using two Viscogel columns, a G4000 PWXL and a G6000 PWXL (the molecular weight ranged from 2 kDa to 8000 kDa) connected to a model Viscotec refractive-index detector at a flow rate of 1.0 mL/min and a column temperature of 23°C. The eluent was an aqueous solution composed of 0.1 M NaNO 3 at pH 7.4. Poly(ethylene oxide) (PEO) standards from Polymer Standard Service, Inc. (Warwick, USA) were used for calibration.

### Chemicals and expression vector

Commercially sourced jetPEI (POLYPLUS-TRANSFECTION Inc, NY, USA), a 22 kDa linear polyethylenimine (PEI), was used according to the manufacturer's protocols. Firefly luciferase cDNA (luc) was subcloned into the pCI-neo Vector, resulting in the construct pCI-neo-luc. Plasmid pCI-neo-luc was transformed into competent Escherichia coli DH5α, and endotoxin-free plasmid DNA was purified using the Qiagen EndoFree Plasmid Max kit (Qiagen, Valencia, CA) according to the manufacturer's instructions. Nanoparticles of DMAEMA/DNA or PEI/DNA were then prepared with different weight ratios and N/P ratios (i.e., ratios of nitrogen atoms on PDMAEMA or PEI to phosphates on DNA).

### Cell culture and animal

BNL 1MEA.7R.1 (chemically-transformed liver cells) were purchased from American Type Culture Collection (Manassas, VA, USA). These cells were grown in Dulbecco's modified Eagle's medium containing 4.5 g/l glucose (DMEM, High Glucose, Gibco, Grand Island, NY, USA), supplemented with 10% fetal bovine serum (Gibco), and a 1% mixture of penicillin G, streptomycin and amphotericin B (Gibco) at 37°C in 5% CO_2_. Male BALB/c mice (18–20g, 6–8 weeks old) were purchased from the Animal Center of National Taiwan University Hospital (Taipei, Taiwan, Republic of China). All experiments were carried out in accordance with the Institutional Animal Care and Use Committee of the National Taiwan University College of Medicine.

### Measurement of zeta-potential and particle size

To determine the size and electrostatic properties of PDMAEMA/DNA nanoparticles, a mixture of PDMAEMA and plasmid DNA was diluted in 150 mM NaCl. Particle size and zeta potential were evaluated using the Nano-ZS (Malvern, Worcestershire, UK). The presented data are the means of at least three measurements.

To evaluate the binding conditions of PDMAEMA and plasmid DNA, plasmid DNA (1 µg) mixed with PDMAEMA at different N/P ratios was incubated at 37°C for 10 min. The mixture was centrifuged at 12000×g and the supernatant (non-binding DNA) was collected and quantified by measuring its absorbance at 260 nm. The binding percentage is defined as: (total amount of DNA–amount of non-binding DNA)/total amount of DNA.

### Ultrasound treatment

#### In vitro study

BNL cells were seeded in 24-well plates at an initial density of 5×10^4^ in 0.5 ml of growth medium and incubated for 24 h prior to US exposure. The 24-well plate was placed above a US probe with a thin layer of US gel in between. Only one well at a time was exposed to US by aligning the center of the exposed well with the center of the US probe. The culture medium contained 1 µg of plasmid DNA with or without a predetermined amount of PDMAEMA (weight ratios of 0.25, 0.5, and 1) or with PEI (N/P ratio 5). US was generated by a commercial gene transfection device, Sonitron 2000 (Rich-Mar Co., OK, USA), equipped with a 1 MHz probe in an average intensity of 2W/cm^2^, a duty cycle (DC) of 20%. The diameter of the US probe is 10 mm. Following the number of indicated days after US exposure, the cells were stripped from the culture plates and suspended in a luciferase lysis buffer (CCLR, Promega, Madison, WI, USA). The cell suspensions were mixed and then centrifuged at 12,000×g, at 4°C for 10 min. The supernatant was assayed with a luciferase assay substrate kit (Luciferase Assay System, Promega, Madison, USA) and luciferase activity was measured by a microplate luminometer (Iinfinite M200, Tecan, Austria). Luciferase activity was normalized to the protein content of the cells. Protein concentration was determined using the Bio-Rad protein assay (Bio-Rad Laboratories, Hercules CA, USA).

#### *In vivo* study

Mixtures of 10 µg plasmid DNA with or without a predetermined amount of PDMAEMA (weight ratios of 0.25, 0.5, and 1) or PEI (N/P ratios 0.5) were mixed with 30% (v/v) of the microbubble echo-contrast agent (SonoVue Bracco, Milan, Italy) to yield a total volume of 100 µl. The mixture solution was intramuscularly injected into the thigh muscle of 6–8 week old male BALB/c mice. The injection site of each mouse then received 5 min of US exposure. US was generated by Sonitron 2000 and a 1-MHz US probe with an average intensity of 2W/cm^2^, a duty cycle of 20%. On predetermined days following the injection, mice were anesthetized by isoflurane and intraperitoneally injected with 100 µl of luciferin (20 mg/ml). The whole body distribution of luciferase expression was monitored by an IVIS imaging system (Xenogen, Alameda, CA, USA). Luciferase activity was quantitatively measured using Live Image 2.5 software (Xenogen, Alameda, CA, USA). The optimal concentrations of SonoVue providing the best gene expression in the muscle tissue were selected according to the criteria established in our previous study [30].

**Figure 1:**
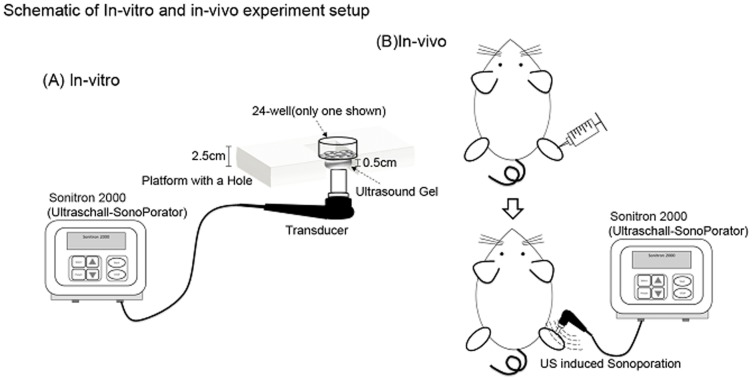
Schematic of *in-vivo* and *in-vitro* experimental setup.

Schematic diagrams of *in-vivo* and *in-vitro* experimental setup are shown in Figure 1.

### Cell viability assay

*In vitro* cytotoxicity tests were performed using an MTT assay. One day after US exposure, the cells were treated with an MTT reagent (Sigma-Aldrich, St. Louis, MO, USA), and were further incubated for 4 h at 37°C. The medium was removed and 1 ml DMSO was added to dissolve the MTT product (formazan crystals). The plate was gently shaken for 15 min to achieve complete dissolution. Aliquots (100 µl) of the resulting solution were transferred to 96-well plates and absorbance was recorded at 570 nm using the microplate spectrophotometer system (Infinite M200, Tecan, Austria). Relative cell viability was calculated as (A_treat_/A _control_)×100%.

### Fluorescence labeling and confocal microscope studies

To investigate how DMAEMA/DNA promoted the transgene expression, the pCI-neo-Luc DNA was labeled using a Fluorescein Label IT Tracker Kit (Mirus Bio Corporation Madison, WI, USA) according to manufacturer's instructions. Subsequently, the fluorescein-labeled DNA was used to prepare PDMAEMA/DNA nanoparticles at a weight ratio of 10 or PEI/DNA nanoparticles at an N/P ratio of 5. The fluorescein-labeled PDMAEMA/DNA or PEI/DNA nanoparticles were added to cultured BNL cells (5×10^4^ cells/0.5 ml) and incubated in 24-well plates for 24 h. One day following US exposure, the exposed cells were rinsed with a PBS solution and cultured in 0.5 ml of fresh culture medium. The intracellular fluorescence change of the transfected cells was monitored on the 2^nd^ and 4^th^ days following US treatment. Each time, the cells were washed twice with ice-cold PBS and fixed with 4% formaldehyde in covered glasses. Cells were then mounted with a Fluoromount-G mounting medium (Southern Biotechnology Associates, Birmingham, AL, USA) and observed using a confocal microscope (Leica TCS SP5 Confocal Spectral Microscope Imaging System, Germany).

### Quantification of DNA uptake

To measure DNA uptake, pCI-neo-Luc DNA was labeled by FITC and then mixed with DMAEMA/DNA nanoparticles at a weight ratio of 10 or PEI/DNA nanoparticles at an N/P ratio of 5. The PDMAEMA/DNA or PEI/DNA nanoparticles were transfected to BNL (5×10^4^ cells/0.5 ml) by US and incubated in a 24-well plate for 24 h. Intracellular uptake of DNA was measured by a LSRII flow cytometer (BD Biosciences, San Jose, CA, USA) and analyzed using FlowJo software (Treestar Software, San Carlos, CA, USA).

### Statistical analysis

All data are presented as the mean ± SEM. The differences were analyzed using the student's t-test or one-way ANOVA. “P” <0.05 was considered statistically significant.

## Results and Discussion

### Physicochemical characterization of PDMAEMA/DNA nanoparticles

**Figure 2:**
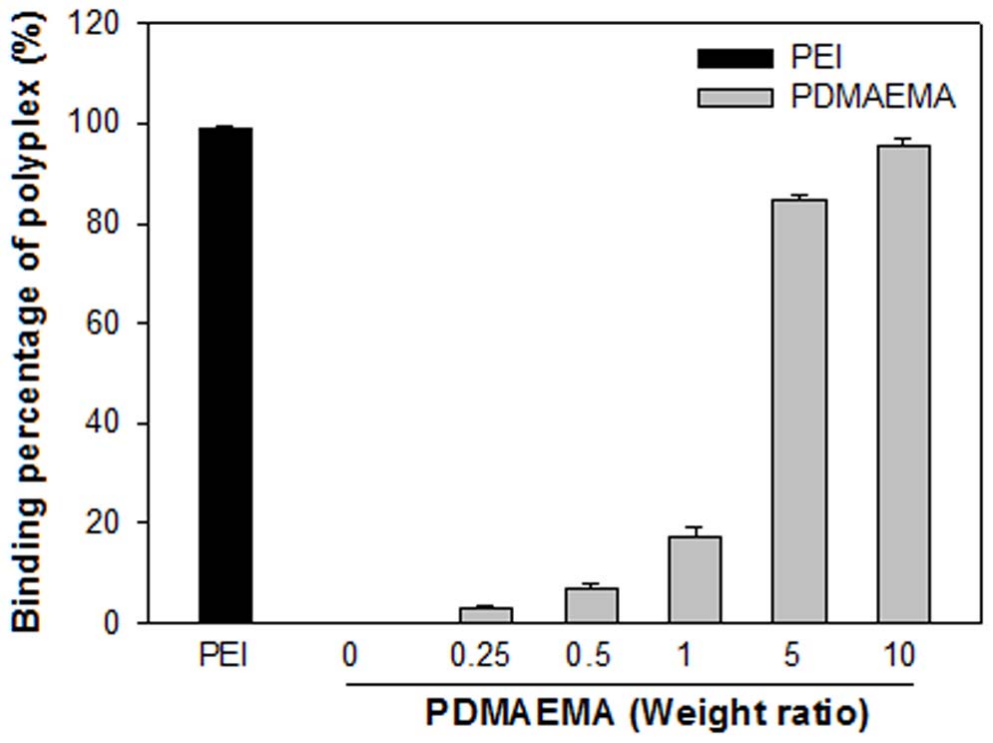
Binding ability of DNA by PDMAEMA at different weight ratios. The binding of DNA saturated at weight ratios of 5 and 10 where no free form DNA remains.

To understand the physicochemical properties of the PDMAEMA/DNA nanoparticles used in all experiments, their particle sizes and zeta-potentials were evaluated and compared. Mixing cationic PDMAEMA with negatively-charged DNA induced the spontaneous electrostatic formation of stable nanoparticle complexes. Table 1 shows the average sizes and zeta potentials for particles formed at a constant DNA concentration of 1 µg. The mean particle size is significantly bigger at a weight ratio of 1, but not significantly different at weight ratios of 0.25, 0.5, 5 and 10. PDMAEMA/DNA nanoparticles are negatively charged at weight ratios of 0.25, 0.5 and 1, but positively charged at N/P ratios of 5 and 10. As shown in Figure 2, the binding percentage of PDMAEMA and plasmid DNA increased with the weight ratio, especially at weight ratios of 5 and 10. This suggests that most plasmid DNA was captured by PDMAEMA to form nanoparticles at weight ratios above 5. No free form plasmid DNA remained.

**Table 1:** Particle sizes and Zeta potentials of PDMAEMA/DNA nanoparticles with different weight ratios.

weight ratios (PDMAEMA/DNA)	Particle size (nm)	Zeta potential (mV)
0.25	173.1±10.3	−33.4±1.7
0.5	200.2±24.3	−35.0±1.0
1	1143.5±91.5	−20.8±1.2
5	264.1±17.6	22.4±0.8
10	146.8±10.4	18.8±0.3

Values are mesn ± SEM for three to four independent messurements.

### Gene expression and cytotoxicity

**Figure 3:**
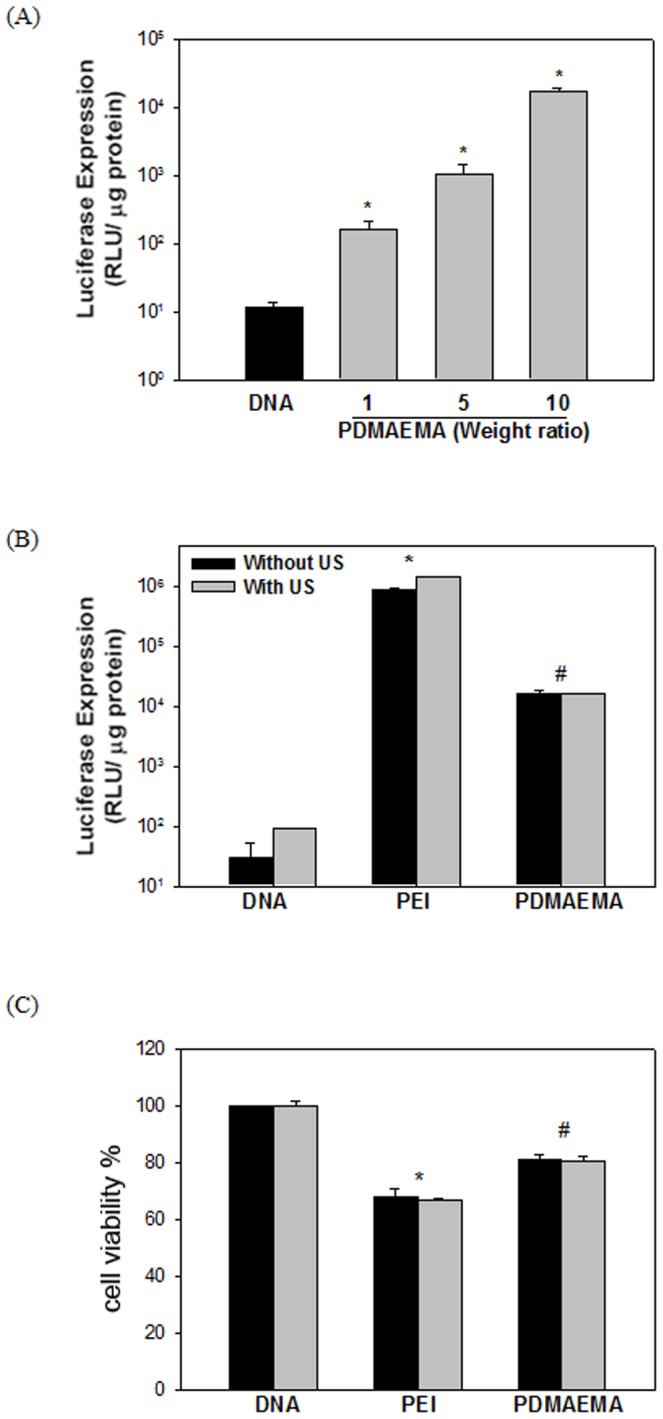
Transgene expression following *in vitro* PEI/DNA and PDMAEMA/DNA with US exposure. (A) One μg DNA was gently mixed with PDMAEMA with different weight ratios (1, 5, 10) or PEI (N/P ratio: 5) at room temperature and incubated for 10 min to form nanoparticle complexes, (B) which were then added to cultured cells and transfected by sonoporation. Luciferase expression was evaluated and compared. (C) Cell viability was assessed by MTT assay 24 h following US treatment and compared with no US condition. All results are expressed as the mean ± SEM for six independent experiments. *^#^ P*< 0.05 vs. PEI or PEI with US; *P<0.05 vs. DNA or DNA with US.

Polyethylenimine (PEI) is known to facilitate both *in vitro* and *in vivo* gene transfection [16], [17]. Thus, the transfection ability of a commercially-sourced linear PEI (22 kDa), jetPEI (POLYPLUS-TRANSFECTION Inc, NY, USA) in BNL cells was compared with that of the PDMAEMA. Experiments were performed at a constant DNA volume (1 µg) mixed with PEI at an N/P ratio of 5 or PDMAEMA at weight ratios of 1, 5, and 10. As shown in Figure 3A, the expression level of luciferase increases with the weight ratio. In Figure 3B, PDMAEMA of a weight ratio of 10 was compared with PEI (N/P ratios: 5) with or without US. The luciferase expression of PEI was significantly higher than that of the PDMAEMA regardless of US exposure (Figure 3B). Cell viability decreased for both the PEI/DNA and PDMAEMA/DNA groups, but PDMAEMA was less toxic than PEI (Figure 3C). Both the PDMAEMA and PEI groups show orders-of-magnitude improvement to transfection ability over the groups with plasmid DNA alone.

### PDMAEMA prolonged the expression duration of US-mediated gene transfection

**Figure 4:**
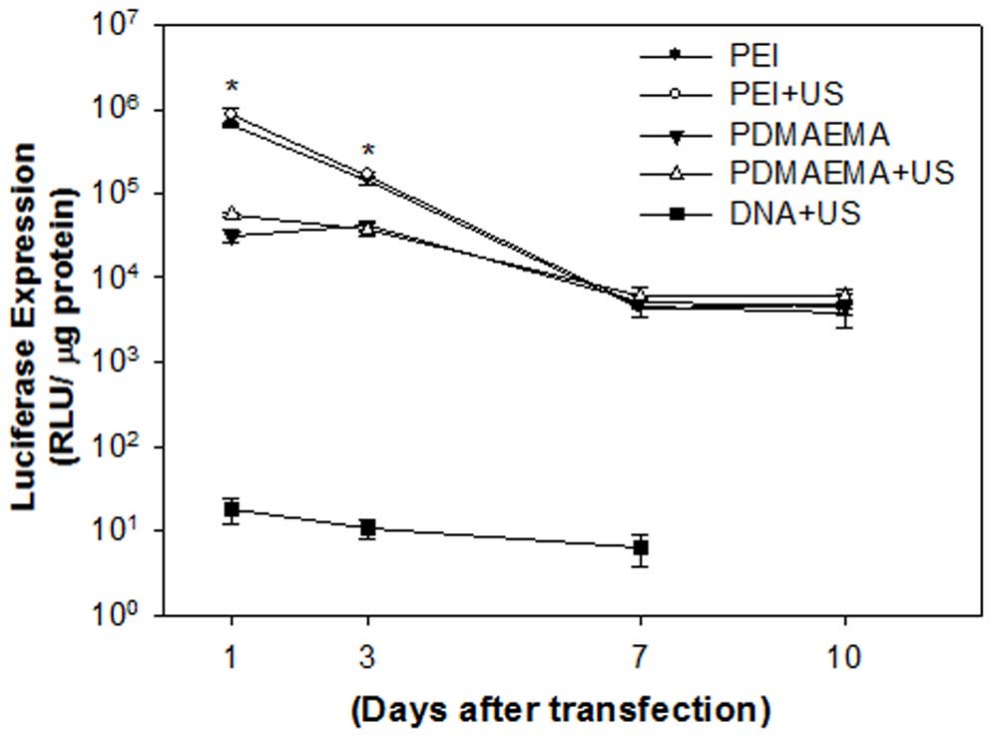
*In vitro* transgenic expression of PEI/DNA nanoparticles at an N/P ratio of 5 and PDMAEMA/DNA nanoparticles at a weight ratio of 10 with or without US exposure. The PEI mediated gene expression on day 1 was significantly higher than that of PDMAEMA regardless of US exposure. However, there was no difference after 7 days. *P<0.05 vs. PDMAEAM.

The level of gene expression was evaluated by transfecting the mixture of 1((g plasmid DNA with PEI or PDMAEMA. As shown in Figure 4, the levels of in-vitro gene expression obtained by PEI with or without US are an order of magnitude higher than those obtained by PDMAEMA regardless of US exposure on Day 1 and Day 3. After seven days, the levels of luciferase intensity for PEI groups decreased and showed no significant difference to the PDMAEMA groups. This result suggests that, for in vitro condition, PEI and PDMAEMA had comparable efficiency for gene transfection regardless of US exposure. However, without the help of PEI or PDMAEMA, the expression levels of luciferase for naked DNA were less than 102 RLU. In this study, PDMAEMA-incorporated DNA complexes with polycation molecular weights ranging from 1.9 kDa (12 repeat units) to 6.1 kDa (39 repeat units) were investigated for gene delivery. Preliminary studies showed that, with its relatively low molecular weight, PDMAEMA was less effective as a gene delivery vector (data not shown). Due to the low positive charge of its polymer chain, low molecular weight PDMAEMA is incapable of effectively condensing DNA. The transfection ability of polyplexes at different weight ratios of nanoparticles to DNA could also be explained by the abovementioned charge theory. In the *in vitro* studies shown in Figure 2, PDMAEMA with higher weight ratios (5 and 10) bind more DNA. This finding supports our previous *in vitro* studies on PEI [25]. Higher DNA binding ability secured higher *in vitro* transgene expression (Figure 3A). In addition, the best binding ability of DNA by low molecular weight PDMAEMA(1.9 kDa, 12 repeat units) is less than 80% (data not shown). On the other hand, the binding ability of DNA by PEI at an N/P ratio of 5 and PDMAEMA at a weight ratio of 10 is almost 100% (Figure 2). PEI/DNA at an N/P ratio of 5 and PDMAEMA/DNA at a weight ratio of 10 also provide the best transfection efficiency and expression duration (at least 10 days) for *in vitro* gene transfection (Figure 4).

**Figure 5:**
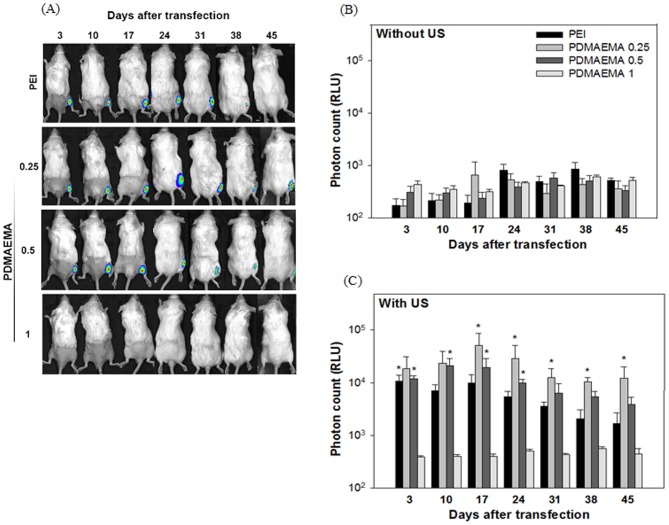
Expression duration of *in vivo* transfected plasmid DNA. DNA (10 µg) was gently mixed with PEI at an N/P ratios of 0.5 or PDMAEMA at weight ratios of 0.25, 0.5 and 1. DNA/PEI and PDMAEMA complexes were then injected into the thigh muscle of mice and transfected by sonoporation. Luciferase activity in Balb/C mice was measured by an IVIS system on gene transfection with (right leg) or without (left leg) US exposure. (A) Quantification of the signal produced in mice muscle after transfection of each formulation (B) without and (C) with US exposure. All results are expressed as the mean ± SEM for five independent measurements (n = 5). *P<0.05 vs. PDMAEAM 1.

To compare the gene expression obtained using PEI and PDMAEMA in mice, we mixed 10((g DNA and SonoVue (30% v/v) with different concentrations of PDMAEMA to obtain weight ratios of 0.25, 0.5 and 1, or with PEI with an N/P ratio of 0.5, before injecting the mixture into the thigh muscle of mice. The injection site was then exposed to 1 MHz, 2 W/cm2 US for 5 min. In-vivo luciferase activity was measured by an IVIS system 3, 10, 17, 21, 28, 35 and 42 days following US exposure. As shown in Figure 5B, without US exposure the level of luciferase activity was not significantly different for all groups, and was close to the noise level (<103 RLU). However, the level of luciferase activity was significantly higher in the US exposure groups, especially at the weight ratios 0.25 and 0.5 for the PDMAEMA groups and for the PEI group (Figure 5C). Moreover, the gene expression using PEI/DNA at an N/P ratio of 0.5 and PDMAEMA/DNA at weight ratios of 0.25 and 0.5 in mice followed a similar pattern, all showing long expression durations and stable gene expression. PDMAEMA with a weight ratio of 0.25 exhibited the best efficiency among all groups, and maintained a relatively stable expression for at least 45 days *in vivo*. For all experiments, adding PEI and PDMAEMA did not reduce mouse survival. It is well known that plasmids transfected using non-viral methods, including US, are only transiently expressed. The expression generally peaks between 4–7 days after transfection and, with rare exceptions, is essentially gone by day 21[7], [8]. For example, the transgene expression of luciferase plasmid in the posterior heart after US-mediated transfection peaked at day 4 but dropped to less than one tenth of the peak value by day 14[15]. In one case, the half period of luciferase expression after gene transfection with bubble liposomes and US in mice was only 0.54 days and, by day 7, the luciferase expression was less than 1% of that on day 1[16]. For our *in vivo* studies, the US-facilitated groups of PEI at an N/P ratio of 0.5 and PDMAEMA at weight ratios of 0.25 and 0.5 all show peak expression around day 17. The expression levels dropped thereafter but were still relatively high compared with groups without the US exposure (Figure 5), which are within the range of background levels. The PDMAEMA group at a weight ratio of 0.25 exhibits the best and most stable expression. The expression levels from day 31 to 45 are statistically identical. Recent research in cancer gene therapy using IL-12 gene concluded that a better vector for delivering IL-12 for treating liver cancer requires low immunogenicity and the ability to produce sustained protein expressions [31]. This long and stable expression ability is an important and powerful feature for a non-viral gene transfection system. The crucial role of US in transfecting polyplexes (PEI/DNA or PDMAEMA/DNA) into muscle tissue is also unique. However, the condition for *in vivo* studies was reversed. Relatively lower weight ratios of PDMAEMA/DNA at 0.25 and 0.5 provide significantly better expression than the higher ratio of 1.0 for *in vivo* studies, and the 0.25 group shows the best and the most stable transgene expression (Figure 5). The *in vivo* findings of PDMAEMA coincided with our previous reports of PEI/DNA complex [25]. As shown in Table 1, the zeta potentials of PDMAEMA/DNA nanoparticles at weight ratios of 0.25 and 0.5 are negative and their particle sizes are around 200 nm. In addition, PDMAEMA at weight ratios of 0.25 and 0.5 had the lowest binding ability of DNA (Figure 2). Therefore, the optimal weight ratio for *in vivo* studies may be attributable to the necessarily negative and small PDMAEMA/DNA nanoparticles. Due to the low surface charge of PDMAEMA/DNA complexes at low weight ratios, these complexes are not able to induce effective cellular uptake through charge-mediated interactions. Therefore, US exposure was required for effective PDMAEMA/DNA transfection in skeletal muscle (as well as for PEI/DNA). Unlike the conditions at weight ratios of 0.25 and 0.5, we cannot find gene expression at a weight ratio of 1 for *in vivo* studies (Figure 5). Although the zeta potential of PDMAEMA/DNA nanoparticles at a weight ratio of 1 is negative, the mean particle size of PDMAEMA is over 1000 nm (Table 1) which may also be responsible for the ineffective *in vivo* transfection. A quantitative *in vitro* study of cellular uptake of different sizes shows that the cellular uptake dramatically decreases with increased nanoparticle size (200, 500 nm) [32].

### PDMAEMA protects DNA against degradation

**Figure 6:**
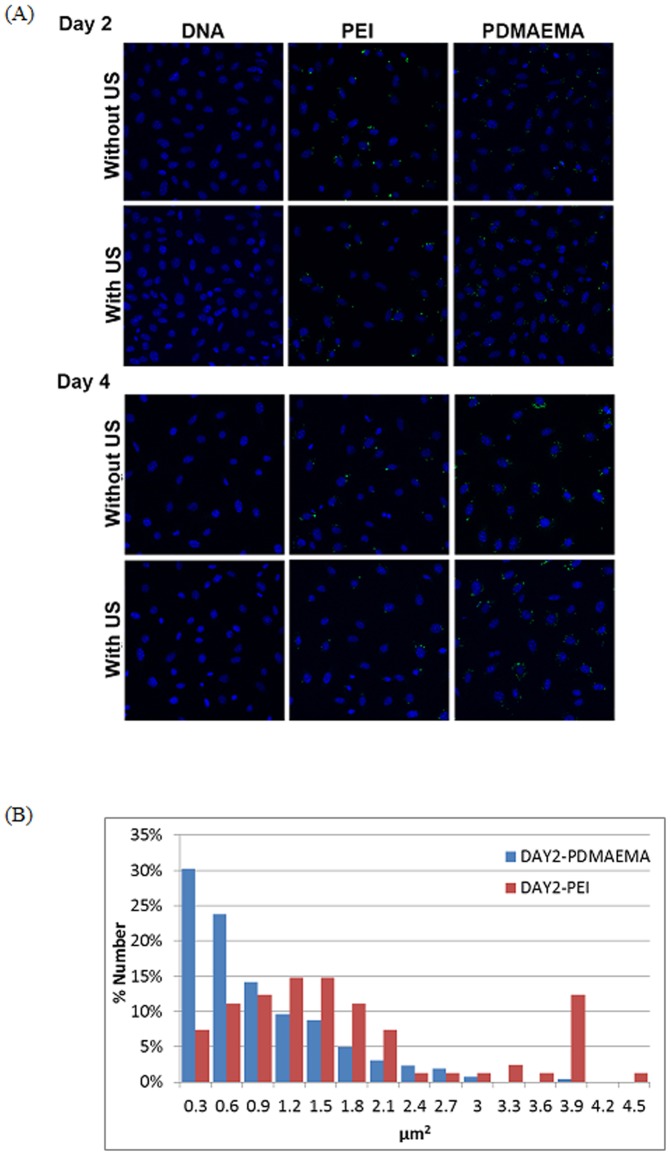
Confocal images of fluorescence labeled DNA for BNL cells treated with DNA, DNA/PEI and DNA/PDMAEMA with or without US exposure. Green: Fluorescein labeled DNA and Blue: DAPI-stained nucleus. (A) The cells were transfected for 24 h and further incubated for 2 days and 4 days. (B) Quantification of the size distribution of transfected PEI/DNA and PDMAEMA/DNA nanoparticles.

Figure 6 shows confocal microscopy images of the cellular uptake of the fluorescein-labeled plasmid DNA. Internalized DNA in PEI and PDMAEMA groups with or without US could all be observed on Day 2 and Day 4 (Figure 6A) following transfection. However, plasmid DNA could not be detected in the naked DNA group on Day 2 and Day 4 regardless of US exposure (Figure 6A). Both PDMAEMA and PEI could enhance DNA internalization and prolong the duration of gene expression by prolonging the lifetime of the intracellular plasmid DNA. However, differences still exist. As shown in Figure 6B, the size distributions of PEI/DNA and PDMAEMA/DNA nanoparticles are very different. The intracellular PEI/DNA nanoparticles are larger in size, and a solitary peak of 3.9 µm^2^ was found in the PEI/DNA group.

### Amount of intracellular uptake of DNA

**Figure 7:**
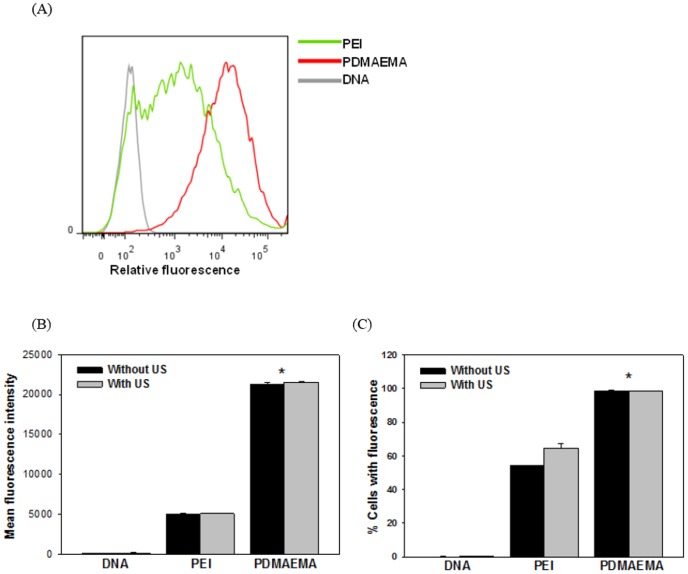
Effect of PEI and PDMAEMA on intracellular uptake of DNA monitored by flow cytometry 24 h following exposures. Fluorescence labeled DNA was mixed with PEI or PDMAEMA in culture cell plates before being treated with US. (B) The overall fluorescence intensities of cultured cells and (C) the percentage of cells with fluorescence are also shown. All results are expressed as the mean ± SEM for four independent measurements. *P<0.05 vs. PEI group (regardless of US exposure).

To further understand the difference between PEI and PDMAEMA in terms of plasmid DNA uptake, the fluorescence-labeled PEI/DNA complexes transfected into BNL cells were quantified by flow cytometry 24 h following the initiation of the transfection process (Figure 7). As shown in Figure 7B, the mean fluorescence and intensities of the PDMAEMA groups were significantly greater than that of the PEI group. PDMAEMA induced more plasmid DNA to enter cells than did PEI. Moreover, significantly more cells in the PDMAEMA groups were found to have internalized the labeled complexes (regardless US exposure) than in the PEI group (Figure 7C). However, it is worth noting here that the overall *in vitro* transfection efficiency of the PDMAEMA/DNA groups is lower than that of the PEI/DNA groups (Figure 3A). The initial *in vitro* expression level of transfected PDMAEMA/DNA is lower than that of the transfected PEI/DNA complexes (Figure 4). The exact reason for the high transfection efficiency but low expression ability of PDMAEMA/DNA as compared to the PEI/DNA complexes is still unclear. The jetPEI used here has secondary amine groups, while PDMAEMA is composed of tertiary amine groups. Is the difference due to differences in the functional groups? Moreover, we found that the intracellular particle size of the PDMAEMA/DNA complexes is smaller than that of the PEI/DNA complexes (Figure 6). The inefficiency may be due to the tight condensation and thus difficult intracellular DNA release of the PDMAEMA/DNA complexes. Thus, a different PDMAEMA polymer having both favorable transfection and expression abilities would be desirable, and further studies are needed to address these questions. PDMAEMA enhanced the US transfection efficiency rate by increasing both the intracellular uptake of plasmid DNA and the percentage of transfected cells (Figure 7). According to the results shown in Figure 7, significantly improved DNA internalization efficiency was found for the PDMAEMA/DNA groups, both in terms of amount per cell and the number of cells. Previous research has shown that the transfection efficiency and cytotoxicity of PEI are largely influenced by its molecular weight, degree of branching, zeta potential and particle size. Many studies have shown that the most suitable molecular weight of PEI for gene delivery ranges from 5 to 25 kDa [33]–[36]. PEI with increased molecular weight exhibits high transfection and expression efficiency partly because the high cationic charge density efficiently condenses the negatively charged DNA into small complexes and protects it from nuclease degradation [37]. However, cytotoxicity has also been found to increase concurrently [36]. PEI with a higher molecular weight has higher cytotoxicity due to cell-surface aggregation of the polymer [38]. Though PEI with a lower molecular weight is less toxic, it does not display the effective transfection properties as a gene delivery vector. The low amount of positive charges within one molecule makes it difficult for the small PEI particles to tightly condense the negatively charged DNA, making it impossible for them to induce cellular uptake through charge-mediated interactions [36].

The key factor in the success of gene therapy is the use of an efficient gene delivery vector. Previous studies have shown that US can enhance gene transfection efficiency by directly delivering DNA into the cytoplasm [10]–[13]. PEI is one of the most successful and widely studied gene delivery polymers, providing good DNA binding and highly effective protection for DNA from nuclease attack [24], [39], which prolongs the expression of the transfected DNA [25]. The present study demonstrates that, following exposure to 1-MHz pulsed US, PDMAEMA/DNA could also significantly enhance the transfection efficiency and prolong the expression duration with minimal toxicity, especially *in vivo*. The use of skeletal muscle in our *in vivo* study has several unique advantages. Muscle constitutes about 30% of adult human body mass and has an abundant blood supply, lending itself to use as good target tissue for the production of secreting transgene proteins to act as systemic therapeutic agents [12], [40], [41]. Moreover, the dosage control may be considerably improved through multiple transfection instances and sites on muscle, showing great potential for future clinical applications.

## Conclusions

Our results show that, similar to linear PEI, PDMAEMA with tertiary amine groups is a potential polycationic carrier for facilitating gene transfection. Aside from enhancing the transfection efficiency, PDMAEMA could significantly prolong the expression duration of transfected reporter genes. US exposure is essential for *in vivo* gene transfection to mouse muscle tissue. PDMAEMA also provides better DNA internalization ability and less toxicity. Low weight ratios (0.25 and 0.5) of PDMAEMA to DNA provide better transfection efficiency for *in vivo* systems.
